# Smartphone Use and Security Challenges in Hospitals: A Survey among Resident Physicians in Germany

**DOI:** 10.3390/ijerph192416546

**Published:** 2022-12-09

**Authors:** Judith Kraushaar, Sabine Bohnet-Joschko

**Affiliations:** Chair of Healthcare Management and Innovation, Faculty of Management, Economics and Society, Witten/Herdecke University, 58455 Witten, Germany

**Keywords:** mobile devices, smartphones, hospitals, residents, information security, apps, working conditions, organization, digitization

## Abstract

Although mobile devices support physicians in a variety of ways in everyday clinical practice, the use of (personal) mobile devices poses potential risks for information security, data protection, and patient safety in hospitals. We used a cross-sectional survey-based study design to assess the current state of smartphone use among resident physicians in hospitals and to investigate the relationships between working conditions, current smartphone usage patterns, and security-related behavior. In total, data from 343 participating physicians could be analyzed. A large majority (98.3%) used their smartphones during clinical practice. Of the respondents who used a smartphone during clinical practice, only 4.5% were provided with a smartphone by their employer. Approximately three-quarters of the respondents who used their smartphones for professional communication never/almost never used dedicated GDPR-compliant messenger services. Using a hierarchical regression model, we found a significant effect of the organizational resources Social Support (Supervisor) and Information Security-related Communication on security-related behavior during the selection of medical apps (App Selection). Smartphones are an important part of digital support for physicians in everyday clinical practice. To minimize the risks of use, technical and organizational measures should be taken by the hospital management, resulting, for example, in a Bring-Your-Own-Device (BYOD) initiative.

## 1. Introduction

In addition to high expectations concerning enhanced efficiency and quality in hospitals, increasing multimorbidity and complex clinical pathways require direct, easy, and quick access to and transmission of care-relevant information, independent of time and location. Mobile devices (especially smartphones and tablets) combined with digital applications (apps) already support physicians in hospitals in several ways, as numerous current mobile health studies show [1,2,3,4,5,6,7,8,9,10,11,12,13,14,15,16,17,18,19] (Table A1). The rapid development in the field of mobile health indicates that mobile devices will increasingly be integrated into everyday clinical practice and are becoming essential devices. Furthermore, the current young generation of physicians consists of digital natives (Generation Y) who have been surrounded by digital devices since childhood and who take their private and professional use for granted [20,21].

The use of mobile devices offers a variety of options for physicians to communicate with each other, but also with hospital staff, patients, and professionals in other sectors, e.g., via calls, e-mails, messenger services, or video conferences. In addition, everyday work can easily be organized via mobile devices (e.g., using calendar functions or rosters). Together with apps, they also enable modern medical education and research while documentation and monitoring are other fields of application in clinical practice. Due to their widespread availability and highly developed cameras and screens, mobile devices are often used for clinical photography. They provide opportunities for remote access to electronic medical records and hospital information systems as information can be obtained flexibly (e.g., at home or directly at the point of care). Mobile devices also serve as diagnostic and therapeutic decision-support tools, e.g., when physicians need specific drug information or medical calculations. In addition, there is a unique possibility of integrating different sensors in smartphones and tablets or coupling with them (e.g., temperature, blood sugar), which supports diagnosis and therapy as well as (remote) patient monitoring. Here, their compactness offers a significant advantage in everyday clinical practice compared to large medical measuring devices.

However, despite the evident advantages outlined above, the use of (personal) mobile devices in clinical practice can be associated with high risks for information security, data protection, and patient safety [22,23,24]. In their case studies, Hedström et al. (2011) showed that employees in healthcare organizations are exposed to different value conflicts—e.g., health-care values vs. information security values—which they have to resolve quickly for each situation during their practice [25]. This poses security risks that need to be considered by the clinic management. In hospitals, information security refers to the state of full functionality of all IT systems, processes, and components, which are necessary for optimal patient care, and the protection of all information required for this. Information security must be guaranteed at all times, so continuous monitoring and rapid responses to breaches and attacks are essential. Cyber attacks on hospital information systems are no longer a rarity – a study from 2020 shows how vulnerable the German hospital landscape is to ransomware attacks [26]. Empirical research on information security-related behavior of employees at work and its supporting organizational and individual factors is a relatively new field. Researchers recognized that, in addition to the technical equipment of the organization to increase information security, it is also necessary for the employees to follow security policies and consciously use information technology (compliance) because non-compliance can lead to security breaches with far-reaching consequences for the organization. Hu et al. (2012) analyzed the role of top management, organizational culture, and individual cognitive beliefs on information security-related behavior among alumni of MIS and MBA programs and found significant effects [27]. D’Arcy and Greene (2014) examined the influence of security culture, job satisfaction, and perceived organizational support on security compliance intentions among computer-using professionals and found positive effects for security culture and job satisfaction [28]. Solomon and Brown (2020) could show relationships between organizational culture, information security culture—as an organizational subculture—and compliance. They further argued that goal orientation among employees has a stronger influence on compliance than rule orientation [29].

Since January 2022, all hospitals in Germany are obligated to implement state-of-the-art information security measures. This is intended to avoid disruptions to hospital operations due to system failures and to ensure the availability and security of patient information [30,31]. However, personal mobile devices, especially smartphones, are often overlooked as a relevant IT resource for physicians and are not taken into account when listing and analyzing information security-critical systems and processes in hospitals.

The primary objective of this study was to systematically record and assess the current status of smartphone use in everyday clinical practice by resident physicians in hospitals. In addition, our study aims to contribute to the research on organizational measures that can promote responsible behavior in order to reduce potential risks and enhance security. A second objective was, therefore, to examine the relationships between working conditions, current smartphone usage patterns, and security-related behavior. In doing so, we want to go a step further than the studies listed in Table A1 and identify specific organizational measures to mitigate security risks.

## 2. Materials and Methods

### 2.1. Study Design

We used a cross-sectional survey-based study design. A structured online questionnaire in German was developed in LimeSurvey. The first page contained information on the target group, the research project, the content of the questionnaire, and the estimated processing time. After accepting the privacy policy, participants were taken to the second page with demographic questions. These were followed by the main part including seven sections: (1) Working Conditions, (2) Resilience, (3) Job Satisfaction and Work Engagement, (4) IT Resources, Information Security, and Data Protection, (5) Information Security-related Awareness and Compliance, (6) Technical Affinity and Innovative Work Behavior, and (7) Mobile Device Usage. At the end of the survey, participants had the opportunity to share their comments with us. The sections relevant to this report are explained below.

Working Conditions: To assess working conditions, we used the following scales from the Copenhagen Psychosocial Questionnaire (COPSOQ): Quantitative Demands, Predictability, Role Conflicts, Quality of Leadership, Social Support, Feedback, Sense of Community, Trust and Fairness, and Appreciation. The COPSOQ is an internationally established instrument to measure psychosocial work factors with good to very good validity and reliability for most of its scales [32] In Germany, the third version of the questionnaire, which we used in our study, was published in 2019 [33]. In contrast to the original questionnaire, we divided the Social Support and Feedback scales into two subscales each (supervisor/colleagues) to separate the social support/feedback from supervisors from the social support/feedback from colleagues, which could be rated differently, especially in hierarchical organizations. In addition, we used two scales (Uncertainty, Further Education) and two individual questions on working hours and shifts from the German instrument for stress-related job analysis for hospital physicians (ISAK) [34,35]. We also included four self-developed items regarding IT resources in the hospital because we could not find a suitable scale in the research literature.

Information Security-related Awareness and Compliance: In addition to state-of-the-art technical information security solutions, employees of an organization should be aware of the importance of information security and trained accordingly to behave in a compliant manner. The items we used to assess information security-related awareness, self-efficacy, top management commitment, and compliance are based on the works of Hu et al. (2012) [27], D’Arcy and Greene (2014) [28], Karlsson et al. (2017) [36], and Solomon and Brown (2020) [29]. We adapted the items to the clinical situation. Overall, this resulted in four items on awareness, two on self-efficacy, one on top management commitment, and four on compliance. 

Mobile Device Usage: The items on mobile device usage are based on a systematic literature review in which we analyzed 41 quantitative studies on the use of mobile devices by physicians during clinical practice. The section consists of five subcategories on specific usage patterns (Communication, Organization, Documentation and Monitoring, Diagnostic and Therapeutic Decision Support, and Knowledge Acquisition and Training), one subcategory on Mobile App Selection, and other single items on the private and professional use of mobile devices. With the subcategory Mobile App Selection, we wanted to know to what extent the participants consider security-related criteria (patient safety, data protection, and information security) when selecting a new app to support diagnosis and therapy.

All English scales and single items were translated into German, checked independently by two bilinguals, and then adapted based on their comments. We used five-point Likert scales ranging from “Never” to “Several times a day”, “Never/almost never” to “Always”, and “Strongly disagree” to “Strongly agree”, respectively. In the Mobile App Selection scale, we added “No experience with such apps” as a sixth possible answer. To ensure content validity, the survey was reviewed by faculty members and statisticians and modified accordingly. It was then piloted with a group of residents, who highlighted and took notes on any remaining ambiguities which we corrected in the final questionnaire. 

### 2.2. Data Collection

Data were collected between March and June 2022. Our target group comprised physicians who are currently undergoing medical specialist training/residency training in hospitals in Germany (henceforth referred to as “residents”). An invitation with a link to the online questionnaire was sent directly to the residents via e-mail or social media channels, or indirectly via our contacts in the medical field. Important contacts were chief physicians, senior physicians, university professors, hospital managers, alumni networks as well as presidents of the German medical societies. In addition, we asked medical experts with significant influence on social media platforms to share the link. The Hartmannbund, an important association of physicians in Germany, forwarded the link to its resident members.

### 2.3. Data Analysis

The data were first exported from Limesurvey to SPSS. Data analysis was performed with IBM SPSS Statistics 28 and only fully completed surveys were included. For all self-developed scales, the dimensionality was controlled via factor analysis using scree plots. Descriptive statistics were used to present the means, standard deviations, and frequencies. We created bar charts to visualize the results of the smartphone usage pattern analysis. Correlation coefficients were then calculated to determine the statistical relationships between smartphone usage patterns and information security-related compliance. Furthermore, a regression analysis was performed to determine the relationship between working conditions and smartphone usage patterns. For all tests, a p value of less than 0.05. was considered statistically significant.

## 3. Results

### 3.1. Preliminary Analyses

A total of 611 people entered the survey, of whom 349 completed it. An exact statement on the response rate cannot be made because we do not have information on the number of residents who received the questionnaire indirectly through our contacts. Data of six participants had to be excluded due to conspicuous response patterns (4×), work in a hospital abroad (1×), and specific information in the comment section (1×). In total, data from 343 participants were included in our analyses.

The factor analysis showed that the items measuring Information security-related Awareness loaded on two factors and we subsequently divided the scale into two scales: Information security-related Awareness and Information security-related Knowledge. Further, based on the factor analysis, we divided the Smartphone Communication scale into three subscales: Communication Channels, Communication Partners (job-related), and Communication Partners (private). To determine the reliability/internal consistency, Cronbach’s alpha/the Spearman–Brown coefficient was calculated for every scale. The two scales Predictability and Communication Partners (private) showed insufficient reliability (<0.7), meaning that their items were not sufficiently related. They were, therefore, excluded from subsequent correlation analyses. We further checked the assumptions of the regression and found no violations.

### 3.2. Sample Characteristics

Table 1 shows the sociodemographic characteristics of the study participants. Almost two-thirds of the participants were female (63.3%). The two age groups with the highest frequency were 31–35 years (40.8%) and 26–30 years (39.1%). There was a total of 16 specialties represented by at least two participants. Most participants were part of a residency program for Internal Medicine (22.2%). This was followed by Surgery (17.2%), Anesthesiology (14.9%), and Pediatrics and Adolescent Medicine (10.8%). Participants were almost evenly distributed across the residency levels, with the fewest residents in their 4th year (15.2%) and most residents in their 5th year or above (29.7%). The majority of the participants worked in a public hospital (61.5%), while approximately one-quarter worked in a non-profit hospital (22.7%) and 13.7% in a private hospital. Most of the hospitals were university/teaching hospitals (80.5%). The size of the hospitals (measured by the number of beds) in which the residents underwent their training varied, whereby most of them worked in a hospital with 300–800 beds (39.1%), followed by hospitals with more than 800 beds (37.6%). Only a few residents had a job in a hospital with less than 300 beds (17.5%).

### 3.3. Smartphone Usage

Almost all of the residents surveyed stated that they used a smartphone for private purposes (99.1%), with 97.1% of them using it several times a day. A large majority of the participants also used their smartphones during clinical practice: in the past six months, 98.3% used their smartphones in at least one of the five categories that we created (Communication, Organization, Documentation and Monitoring, Diagnostic and Therapeutic Decision Support, Knowledge Acquisition and Training). Only 1.7% of the participants always chose “Never” in all five categories and, thus, had never used their smartphone in clinical practice in the past six months. Of those who used a smartphone during clinical practice, only 4.5% were provided with a smartphone by their employer.

#### 3.3.1. Communication

**Figure A1:**
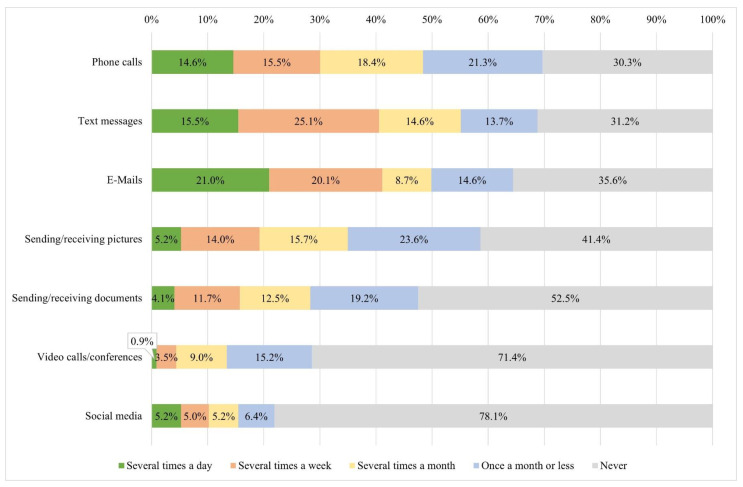
Communication Channels.

**Figure A2:**
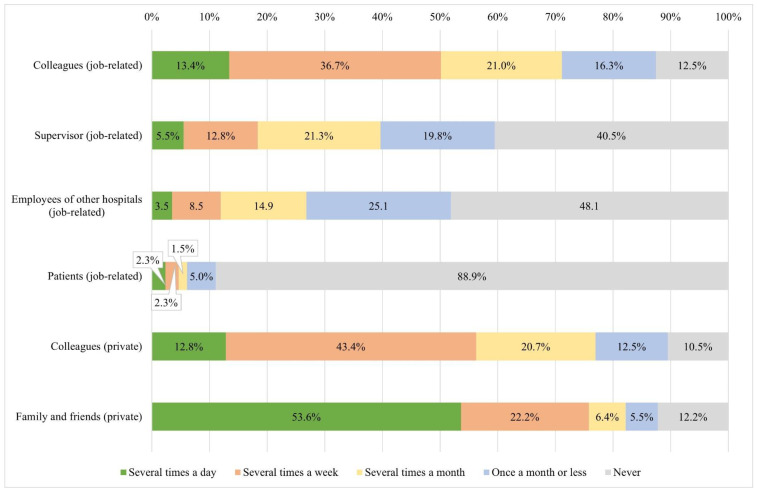
Communication Partners.

During clinical practice, about half of the participants used their smartphones regularly (several times a month or more) for text messages (55.1%), e-mails (49.9%), and/or phone calls (48.4%) (Figure A1). More than a third of the residents regularly received or sent pictures (35.0%), while 28.3% regularly received or sent documents. The majority of the respondents had never made a video call/conference (71.4%) and had never used social media (78.4%) on their smartphones during clinical practice in the past six months. The residents most frequently communicated with colleagues—most of them, several times a month or more for private and/or job-related reasons (77.0%/71.1%) (Figure A2). Smartphones were also regularly used by 39.7% of the participants to communicate with their supervisors. Approximately one-quarter of the respondents frequently communicated with staff at other hospitals. Communication with patients via smartphone was not common and 88.9% had never used it for this purpose. More than three-quarters of the residents surveyed regularly used their smartphones for private communication with their families and friends during clinical practice (82.2%).

#### 3.3.2. Organization

**Figure A3:**
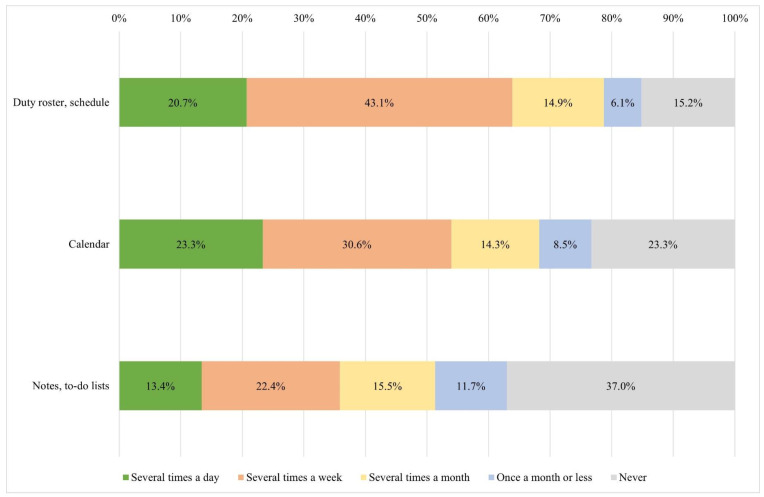
Organization.

Many residents had duty rosters and/or schedules on their smartphones—78.7% used them on a regular basis (Figure A3). The calendar function was also commonly used (several times a month or more) by more than two-thirds of those surveyed (68.2%). Around half of the residents took notes and/or created to-do lists on their smartphones several times a month or more (51.3%).

#### 3.3.3. Documentation and Monitoring

**Figure A4:**
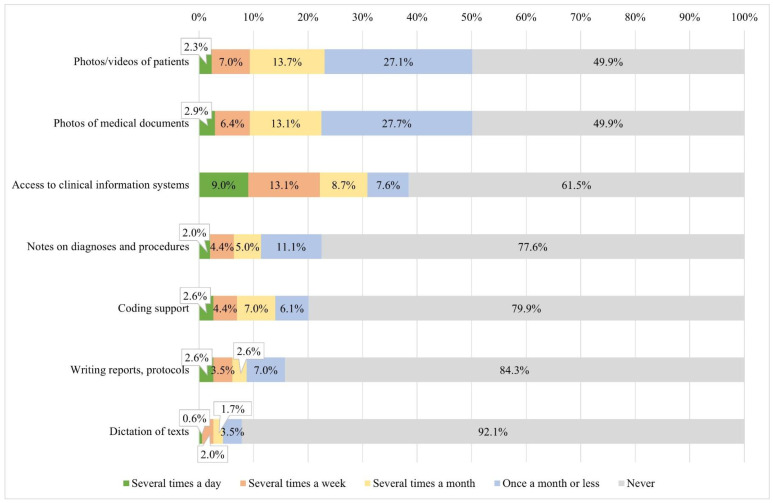
Documentation and Monitoring.

Approximately one-third of the residents regularly accessed clinical information systems via their smartphones (30.9%) and one-quarter commonly took pictures/videos of patients with their smartphones (e.g., to document wounds, injuries, or the course of treatment, or to make before-and-after pictures) (23.0%) (Figure A4). Photographs of medical documents (e.g., X-rays, CT/MRI scans, medical records, laboratory results) were taken with a similar frequency (22.4%). The other four activities surveyed (Notes on diagnoses and procedures, Coding support, Writing reports/protocols, and Dictation of texts) were rarely carried out with the help of smartphones.

#### 3.3.4. Diagnostic and Therapeutic Decision Support

**Figure A5:**
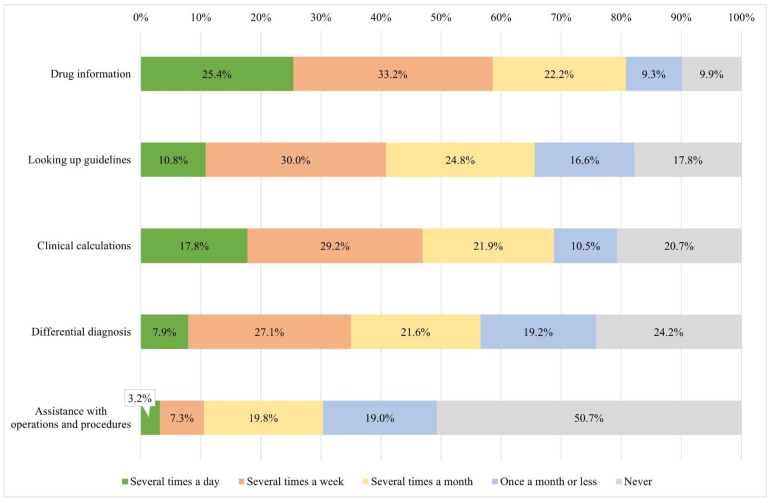
Diagnostic and Therapeutic Decision Support.

In general, activities in this category were often supported by smartphones (Figure A5). A large majority of the participants frequently used their smartphones to search for drug information (80.8%). Approximately two-thirds of the residents performed clinical calculations (e.g., for doses, scores, indices) by using their smartphones several times a month or more (68.8%). About the same number of participants regularly looked up guidelines via smartphone (65.6%) and more than half of the residents used them frequently for differential diagnoses (56.6%). In the past six months, slightly less than half of the respondents had used their smartphones to look for assistance with operations and procedures (e.g., through video tutorials) (49.3%) and almost a third even did so regularly (30.3%).

#### 3.3.5. Knowledge Acquisition and Training 

**Figure A6:**
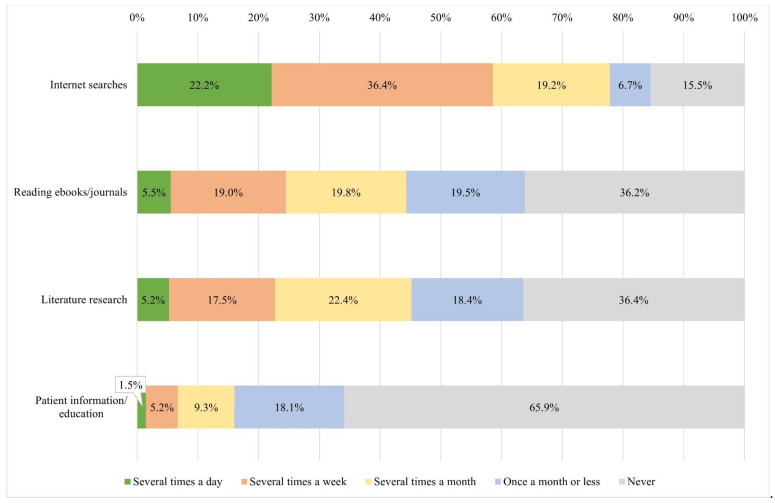
Knowledge Acquisition and Training.

Around three-quarters of those surveyed carried out simple internet searches via smartphone during clinical practice on a regular basis (77.8%) (Figure A6). During clinical practice, 44.3% read e-books on their smartphone several times a month or more and an equal number frequently carried out literature research via smartphone (45.2%). Smartphones were rarely used for patient information/education. In the past six months, 84.0% had used them once a month or less or even never for this purpose. 

### 3.4. Medical App Usage and Selection

**Figure A7:**
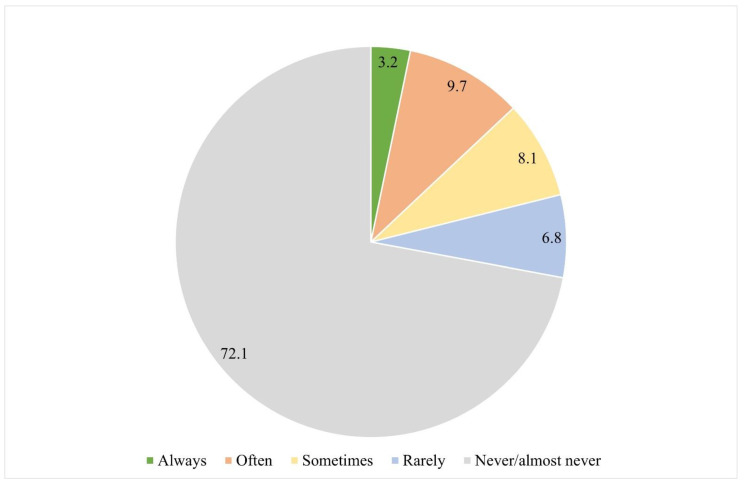
Use of GDPR-compliant Messenger Services.

**Figure A8:**
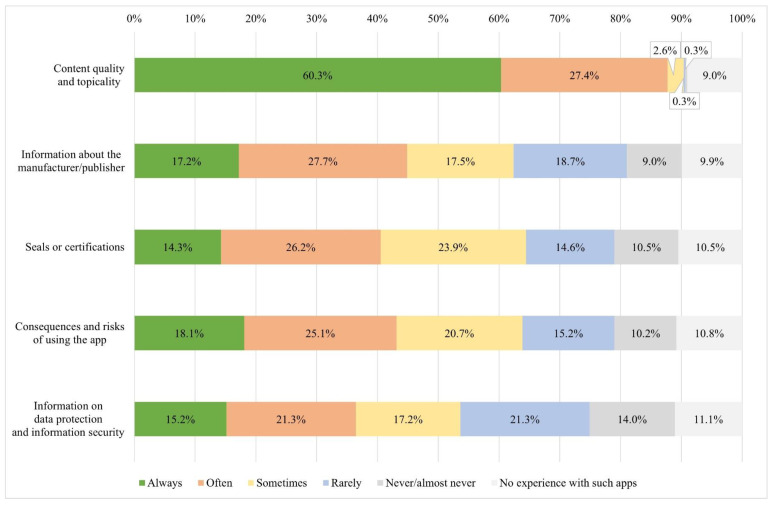
App Selection—Consideration of Security-related Aspects.

Of those who communicated professionally with their smartphone (*N =* 308), 72.1% never/almost never used special, GDPR-compliant messenger services and only 3.2% always used them (Figure A7). Around 90% of the participants already had experience with apps to support diagnosis and therapy. When selecting such apps, most of them often or even always paid attention to content quality and topicality (96.5%) (Figure A8). This result is in contrast to the four other safety criteria that were queried. Here, the proportion of participants with such app experience who often or always considered the respective criterion was below 50%: Information about the manufacturer/publisher (49.8%), Consequences and risks of using the app (48.4%), Seals or certifications (45.3%), and Information on data protection and information security (41.0%).

### 3.5. Relationships between Smartphone Usage, App Selection, and Information Security-Related Compliance

In this section, we wanted to exploratively investigate the correlations between smartphone usage patterns and information security-related compliance, as well as between the consideration of security-related criteria during app selection and information security-related compliance. The strength of the linear correlations was assessed according to Cohen [37].

Table A2 shows an overview of the correlations between the individual variables. There is a weak negative correlation between smartphone use across different communication channels and information security-related compliance (*r =* −0.155, *p <* 0.01). There is also a weak negative correlation between the use of smartphones for documentation and monitoring and information security-related compliance (*r =* −0.189, *p <* 0.01). There are no significant correlations between the other categories of smartphone usage and information security-related compliance. There is a moderate positive correlation (*r =* 0.348, *p <* 0.01) between the consideration of security-related criteria during app selection and information security-related compliance.

### 3.6. Relationship between Organizational Resources and App Selection

In this section, the aim was to analyze whether certain working conditions, especially organizational resources, have an impact on the consideration of security-related criteria when selecting an app for diagnosis and therapy. Specifically, we hypothesized that social support from colleagues and supervisors as well as information security-related communication can predict the consideration of security-related criteria during app selection. This is based on the research model of D’Arcy and Greene (2014) [28].

Table A2 shows that both of the organizational resources Social Support (Supervisor) (*r =* 0.191, *p <* 0.01) and Information Security-related Communication (*r =* 0.167, *p <* 0.01) show a weak positive correlation with the variable App Selection. A significant correlation between Social Support (Colleagues) and App Selection could not be found. There were also weak positive associations between the personal resource Affinity for Technology Interaction (ATI) (*r =* 0.224, *p <* 0.01), measured by the Ultra-Short Scale for Assessing Affinity for Technology Interaction [38], and the Residency Level (*r =* 0.146, *p <* 0.05). Therefore, ATI and residency level will serve as control variables in the following regression analysis.

The hierarchical regression model for predicting safety-related behavior (App Selection) is shown in Table 2. There was a significant effect of the organizational resources Social Support (Supervisor) (*β =* 0.210, *p =* 0.001) and Information security-related Communication (*β =* 0.125, *p =* 0.026) on the dependent variable App Selection. There was no significant effect of Social Support (Colleagues) on App Selection (*β =* −0.069, *p =* 0.282). The control variables Residency Level (*β =* 0.109, *p =* 0.049) and ATI (*β =* 0.215, *p <* 0.001) also had significant effects on App Selection.

## 4. Discussion

This study demonstrates the high prevalence of smartphone use during clinical practice among resident physicians in hospitals in Germany. Well over 90% of the residents surveyed used their smartphone at work, while fewer than 5% received a smartphone from their employer for professional use. These high usage rates of personal smartphones are in line with results of other recent studies on the use of mobile devices in clinical settings, both nationally and internationally [2,3,7,11,16].

Approximately one in two respondents regularly used a smartphone at work to communicate via phone calls, text messages, and e-mails, most frequently with colleagues. Smartphones have great potential to increase the efficiency of communication in hospitals: Compared to one-way pagers and stationary phones, smartphones can have significant advantages in terms of flexibility, reception, and the convenience and efficiency of information transfer. We also asked about the use of smartphones for sending/receiving pictures and medical documents and found that more than one in four participants regularly used their smartphones for this purpose. Other studies found similar results or even higher rates for this aspect, also depending on the respective specialty [3,9,11,12,13]. From a data-protection perspective, this is critical if private communication takes place via the same mobile device, which was the case for most our study participants, as only a few of them were provided with smartphones by their employers. This is also reinforced by the frequent private communication with friends and family, as mentioned by the participating residents. Thus, the great potential of smartphones to improve clinical communication is offset by the risks of data breaches. 

The study shows that smartphones are particularly helpful in organizing day-to-day clinical work. The availability and use of electronic duty rosters and schedules on smartphones appear to be widespread. However, we believe that there is great potential in this field, for example, through intelligent and connected task and workflow management.

Approximately one in three residents regularly accessed clinical information systems with their smartphone as part of documentation and monitoring. The advantages of smartphones here are their high flexibility in terms of location and time of information access. For example, if no digital data are available at the point of care, accessing the clinical information system via smartphone seems to be the most efficient solution. However, there is a particular risk of breaching information security while accessing clinical information systems via smartphones. 

The use of smartphones to support diagnostic and therapeutic decisions was particularly frequent among the respondents in our study. The majority of participants used their smartphones to obtain drug information, look up guidelines, and perform clinical calculations and differential diagnoses. The results are in line with those of most other studies that have investigated this topic in recent years [1,2,3,7,8,9,10,11,14,16,19]. Approximately 90% had experience with apps to support diagnosis and therapy. There is great potential for innovation and digitization in this field. However, users must also be aware of the potential risks for information security, data protection, and, in particular, patient safety. The results of the study regarding the consideration of security-related criteria when selecting an app for therapeutic and diagnostic decision support indicate that some criteria were only taken into account to a limited extent, which could lead to a security gap.

Smartphones have become important devices in everyday clinical practice and can, therefore, be seen as part of the digital transformation process in hospitals. However, our study highlighted potential risks for information security, data protection, and patient safety. Here, we see various ways in which the organization or hospital management can reduce these risks: banning the use of personal smartphones in everyday clinical practice (1), providing hospital-owned devices (2), the prevention and rapid detection of breaches with state-of-the-art IT (security) equipment (3), and training and supporting employees on security-related topics (4). In our opinion, banning the use of smartphones as a measure to increase information security might have detrimental effects on the digital transformation in hospitals and should, therefore, not be considered as a stand-alone policy. Separating private and professional use by providing a hospital-owned smartphone or a dedicated short-range communication device that is capable of performing essential tasks, such as communication, access to clinical information systems, photo taking, or calculating medical scores, could be better options. The usability of hospital-owned devices and accessible applications should be evaluated prior to the hospital-wide implementation to avoid high costs with little benefit. Improving IT and IT security equipment is an important and effective measure, as physicians are currently almost forced to use their own digital devices in everyday clinical practice to work efficiently. Apps for GDPR-compliant communication are used by very few of the participants surveyed. Especially when sensitive patient data are exchanged via smartphone, it is imperative to use GDPR-compliant (medical) messenger services. Some providers now offer a variety of additional functions that could be helpful to physicians, e.g., video calls, case creation, photo editing (e.g., for anonymization), and direct connection to the clinical information system. Their use should be considered by the clinic management. The reasons for the current low usage rates would have to be assessed in a further survey but could be due to low penetration and acceptance. In addition, physicians have to find appropriate apps to support their work themselves. The provision of quality-assured apps for the above-mentioned usage patterns, e.g., by the organization or medical societies, could increase transparency and security.

In addition to technical support, another way to minimize the risks of smartphone use in everyday clinical practice is to train and support employees. We were able to show that social support from supervisors and information security-related communication correlate positively with the consideration of security-related criteria when selecting an app for diagnosis and therapy support. We hypothesize that social support from supervisors may improve residents’ job satisfaction and their sense of responsibility, which, in turn, increases compliance. Information security-related communication could provide greater awareness of possible information security-related risks and, thus, lead to greater compliance so that security-related aspects are given more consideration. Both technical and organizational support could then culminate in a Bring-Your-Own-Device (BYOD) initiative. For the secure integration of personal mobile devices into everyday clinical practice, a BYOD initiative should represent a complex network of technical and organizational measures [22,39,40].

A few limitations should be taken into account when considering and evaluating our results, one of which pertains to the study design. First, since our study was conducted in Germany, it is not possible to directly apply the results to other countries. However, our study is intended to create incentives to conduct studies in hospitals in other countries to explore organizational factors for improving safety-related behavior when using mobile devices. Second, when using an online survey, comprehension problems on the part of the participants cannot be identified and addressed. However, we believe that the approach was the most suitable for the aim of the study and the selected target group as, for example, interviews would have resulted in a much smaller sample and reduced the validity of the results. Third, the cross-sectional design does not allow us to form causal relationships between the organizational factors and the behaviors, but only correlations. Any alterations in physicians’ behavior due to changes in organizational factors can only be assessed with a longitudinal design. 

Even though it was an anonymous online questionnaire, the risk of social desirability bias remains, with participants trying to be much more positive about their actual smartphone use behavior. Specifically, concerning risky behaviors (e.g., sending patient images/data), such a bias might have arisen.

Furthermore, the fact that we cannot precisely determine the response rate represents another limitation. Since we do not have information on the number of residents who received the questionnaire indirectly through our contacts e.g., via chief physicians or the hospital management, we are not able to make an exact statement on the number of residents being invited, which is the basis for calculating the response rate. However, based on the information available to us, we estimate that fewer than 10% of those who received the questionnaire actually responded. If there are systematic differences between the responders and non-responders, the results of our survey may not be representative of the target population. This so-called non-response bias may threaten the external validity of our study by reducing the representativeness of the results. However, previous research suggests that physician surveys are less susceptible to non-response bias than general population studies because they are a more homogeneous study population [41]. Moreover, it is not always the case that a low response rate automatically reduces the representativeness, which is why the response rate should not be considered in isolation [42]. We believe the main reason for the low response rate was the heavy workload, which did not allow physicians the time to participate. Another indicator for the representativeness of a study is the sampling method [43]. Our study may have appealed to physicians with a higher average digital affinity than in the target population. To sum up, statistical conclusions on the entire target population should, therefore, always be drawn taking into account the supposedly limited representativeness.

## 5. Conclusions

To our knowledge, this is the first study to present findings on residents’ smartphone use in hospitals and its association with security-related behavior. Smartphone use in clinical practice was very common among resident physicians in hospitals and smartphones were only rarely provided to the physicians surveyed by their employers. Instead, most of them used their own devices for a variety of different activities. This poses potential risks to information security, data protection, and patient safety in hospitals. These can potentially be reduced through the appropriate use of organizational resources. Here, our results show that organizational measures correlate significantly with security-related behavior and might be able to influence it positively. 

In particular, it is a matter of adequate training and information for the employees, i.e., creating awareness of potential benefits, and innovative applications, but also for potential risks of smartphone use during clinical practice and, at the same time, integrating state-of-the-art IT and IT security equipment. These aspects, when combined in a complex BYOD initiative, could subsequently improve information security, data protection, and patient safety.

## Figures and Tables

**Table 1:** Sample characteristics.

Variable	*n*	%
**Gender**		
Female	217	63.3
Male	125	36.4
Other	1	0.3
**Age group (years)**		
21–25	7	2.0
26–30	134	39.1
31–35	140	40.8
36–40	37	10.8
40 or older	25	7.3
**Specialty**		
Internal Medicine	76	22.2
Surgery	59	17.2
Anesthesiology	51	14.9
Pediatrics and Adolescent Medicine	37	10.8
General Medicine	20	5.8
Neurology	19	5.5
Gynecology and Obstetrics	18	5.2
Radiology	13	3.8
Psychiatry and Psychotherapy	11	3.2
Neurosurgery	8	2.3
Child and Adolescent Psychiatry and Psychotherapy	7	2.0
Urology	5	1.5
Ophthalmology	5	1.5
Otorhinolaryngology	3	0.9
Pathology	2	0.6
Psychosomatic Medicine and Psychotherapy	2	0.6
Other	7	2.0
**Residency level**		
1st year	67	19.5
2nd year	64	18.7
3rd year	58	16.9
4th year	52	15.2
5th year or higher	102	29.7
**Hospital sponsorship**		
Private	47	13.7
Public	211	61.5
Non-profit	78	22.7
I don’t know	7	2.0
**University/teaching hospital**		
Yes	276	80.5
No	62	18.1
I don’t know	5	1.5
**Hospital size (beds)**		
Less than 300	60	17.5
300–800	134	39.1
More than 800	129	37.6
I don’t know	20	5.8

Note. *N =* 343.

**Table 2:** Hierarchical Regression Analysis to predict security-related behavior (App Selection).

Variable	$\Delta R^{2}$	*β*	*SE*	95% CI		*p*
				LL	UL	
1	0.064					
(Constant)			0.190	2.281	3.030	<0.001
Residency Level		0.119	0.032	0.005	0.129	0.035
ATI		0.217	0.042	0.080	0.246	<0.001
2	0.056					
(Constant)			0.362	1.369	2.794	<0.001
Residency Level		0.109	0.031	0.000	0.123	0.049
ATI		0.215	0.041	0.081	0.243	<0.001
Social Support (Supervisor)		0.210	0.055	0.072	0.290	0.001
Social Support (Colleagues)		−0.069	0.081	−0.246	0.072	0.282
Inform. sec.-rel. Commun.		0.125	0.053	0.015	.224	0.026

Note. *R² =* 0.117. Only respondents who had experience with diagnostic and therapeutic decision support apps were included. *N =* 302. CI = confidence interval; LL = lower limit; UL = upper limit.

## Data Availability

The datasets used and/or analyzed in the context of this study are available from the corresponding author upon reasonable request.

## Appendix A

**Table A1:** Current Mobile Health Studies.

#	Author(s)	Year of Publ.	Country	Medical Specialty	Study Population	# of Particip. Physic.	Types of Mobile Devices	Data about Application Patterns Considered for Prevalence Analysis			
								Comm. & Org.	Doc. & Monit.	Diagn. & Therap. Dec. Supp.	Educat.
1	Al Harrasi et al. [1]	2021	Oman	Cross-specialty	Junior physicians, senior physicians	266	not further specified (“mobile handheld devices”/”POC devices”)	√	√	√	√
2	Hitti et al. [2]	2021	Lebanon	Emergency medicine	Junior physicians, senior physicians, medical students, nurses	42	Smartphones, tablets, smart watch/band	√	√	√	√
3	Jahn et al. [3]	2021	UK, Ireland	Pediatrics	Junior physicians, senior physicians, GPs	198	not further specified (“mHealth use”)	√	√	√	√
4	Al Owaifeer et al. [4]	2020	Saudi Arabia	Ophthalmology	Junior physicians, senior physicians	248	Smartphones, tablets, laptops	No distinction between smartph., tablets, and laptops during the analysis of usage patterns.			
5	Dittrich et al. [5]	2020	Germany	Orthopedics and Trauma Surgery	Junior physicians, senior physicians	206	Smartphones				√
6	Lavorgna et al. [6]	2020	Italy	Neurology	Junior physicians, senior physicians	405	Smartphones, tablets, personal computers	No distinction between smartph., tablets, and personal computers during the analysis of usage patterns.			
7	Maassen et al. [7]	2020	Germany	Cross-specialty	Junior physicians, senior physicians, others	303	Smartphones, tablets	√	√		√
8	Zeiger et al. [8]	2020	USA	Neurology	Junior physicians, senior physicians	213	Smartphones	√	√	√	√
9	Buabbas et al. [9]	2019	Kuwait	Dermatology	Junior physicians, senior physicians	101	Smartphones	√	√	√	√
10	Teferi et al. [10]	2019	Ethiopia	Cross-specialty	Junior physicians, senior physicians, GPs	417	Smartphones		√	√	√
11	Yahya [11]	2019	Nigeria	Cross-specialty	Junior physicians, senior physicians	326	Smartphones	√	√	√	√
12	El Hadidy et al. [12]	2018	Ireland	Cross-specialty (Pediatric surgery)	Junior physicians, senior physicians	132	Smartphones	√	√	√	
13	Kameda-Smith et al. [13]	2018	Canada	Neurosurgery	Junior physicians	76	Smartphones, tablets	√	√		
14	Terry & Terry [14]	2018	USA	Family medicine, internal medicine	Junior physicians	39	Smartphones			√	√
15	Gipson et al. [15]	2017	USA	Psychiatry	Junior physicians	68	Smartphones, tablets	√	√		
16	Nerminathan et al. [16]	2017	Australia	Cross-specialty	Junior physicians, senior physicians	109	Smartphones, tablets, laptops	√		√	
17	Stergiannis et al. [17]	2017	Greece	Cross-specialty	Junior physicians, senior physicians, nurses, nurse assistants	352	Smartphones	No distinction between physicians and nurses during the analysis of usage patterns.			
18	Vallangeon et al. [18]	2017	USA	Pathology	Junior physicians	171	Smartphones, tablets, laptops	√			√
19	Jahanshir et al. [19]	2017	Iran	Cross-specialty	Junior physicians	65	Smartphones			√	√

## Appendix C

**Table A2:** Relationships between study variables.

Variable	*M*	*SD*	1	2	3	4	5	6	7	8	9	10	11	12	13	14
1. Gender ^a^	/	/	—													
2. Residency Level ^b^	/	/	0.024	—												
3. Commun. (channels) ^b^	/	/	0.153 **	0.180 **	—											
4. Commun. (partners, job-rel.) ^b^	/	/	0.072	0.151 **	0.583 **	—										
5. Organization ^b^	/	/	0.122 *	0.058	0.467 **	0.323 **	—									
6. Docum. & Monitoring ^b^	/	/	0.072	0.065	0.469 **	0.405 **	0.341 **	—								
7. Diagn. & Therap. Dec. Supp. ^b^	/	/	0.103	−0.108 *	0.337 **	0.317 **	0.496 **	0.431 **	—							
8. Knowl. Acq. & Training ^b^	/	/	0.194 **	0.039	0.376 **	0.373 **	0.492 **	0.386 **	0.635 **	—						
9. Social Support (Colleagues)	4.19	0.67	−0.011	−0.057	0.007	0.016	0.051	−0.085	−0.021	0.037	—					
10. Social Support (Supervisor)	3.53	0.98	0.086	−0.053	−0.022	0.053	0.049	−0.065	0.014	0.068	0.532 *	—				
11. ATI	3.78	1.15	0.407 **	0.018	0.063	0.023	0.211 **	0.062	0.085	0.208 **	−0.031	−0.014	—			
12. Inform. sec.-rel. Commun.	2.69	0.88	0.090	0.110	0.027	−0.026	0.057	−0.040	−0.075	−0.055	0.137 *	0.192 **	−0.013	—		
13. Inform. sec.-rel. Compliance	3.56	0.72	−0.037	0.035	−0.155 **	−0.105	0.097	−0.189 **	−0.014	−0.005	0.131 *	0.193 **	0.085	0.292* *	—	
14. App Selection ^c^	3.49	0.85	0.049	0.146 *	−0.002	0.081	0.125 *	−0.088	0.062	0.077	0.045	0.191 **	0.224 **	0.167 **	0.348 **	—

^a^ Female = 1, Male = 2. *N* = 342. ^b^ Ordinal data. Therefore, Spearman’s Rank Correlation Coefficient was used. ^c^ Only respondents who had experience with such apps were included. *N* = 302. * *p* < 0.05. ** *p* < 0.01. All statistically significant correlations are highlighted in grey.

## References

[1] Al Harrasi A., Al Mbeihsi L.M., Al Rawahi A., Al Shafaee M. (2021). Perception and usage of point of care devices: A cross-sectional study targeting residents and trainers in Oman. Oman Med. J..

[2] Hitti E., Hadid D., Melki J., Kaddoura R., Alameddine M. (2021). Mobile device use among emergency department healthcare professionals: Prevalence, utilization and attitudes. Sci. Rep..

[3] Jahn H.K., Jahn I., Behringer W., Lyttle M.D., Roland D. (2021). On behalf of Paediatric Emergency Research United Kingdom, Ireland. A survey of mHealth use from a physician perspective in paediatric emergency care in the UK and Ireland. Eur. J. Pediatr..

[4] Al Owaifeer A.M., Al Taisan A., Alqahtani B., Alburayk K., Alsubaie M., Alenezi S.H. (2020). Ownership and usage of mobile devices among ophthalmology residents and attending physicians: Identifying the generation gap. Adv. Med. Educ. Pract..

[5] Dittrich F., Back D.A., Harren A.K., Landgraeber S., Reinecke F., Serong S., Beck S. (2020). Smartphone and app usage in orthopedics and trauma surgery: Survey study of physicians regarding acceptance, risks, and future prospects in Germany. JMIR Form. Res..

[6] Lavorgna L., Brigo F., Abbadessa G., Bucello S., Clerico M., Cocco E., Iodice R., Lanzillo R., Leocani L., Lerario A. (2020). The Use of Social Media and Digital Devices among Italian Neurologists. Front. Neurol..

[7] Maassen O., Fritsch S., Gantner J., Deffge S., Kunze J., Marx G., Bickenbach J. (2020). Future mobile device usage, requirements, and expectations of physicians in German university hospitals: Web-based survey. J. Med. Internet.

[8] Zeiger W., DeBoer S., Probasco J. (2020). Patterns and perceptions of smartphone use among academic neurologists in the United States: Questionnaire survey. JMIR mHealth uHealth.

[9] Buabbas A.J., Sharma P., Al-Abdulrazaq A., Shehab H. (2019). Smartphone use by government dermatology practitioners in Kuwait: A self-reported questionnaire based cross-sectional study. BMC Med. Inform. Decis. Mak..

[10] Teferi G.H., Tilahun B.C., Guadie H.A., Amare A.T. (2021). Smartphone Medical App Use and Associated Factors Among Physicians at Referral Hospitals in Amhara Region, North Ethiopia, in 2019: Cross-sectional Study. JMIR mHealth uHealth.

[11] Yahya H. (2019). Healthcare-related smartphone use among doctors in hospitals in Kaduna, Nigeria—A Survey. Niger. J. Clin. Pract..

[12] El Hadidy T.S., Alshafei A.E., Mortell A.E., Doherty E.M. (2018). Smartphones in clinical practice: Doctors’ experience at two Dublin paediatric teaching hospitals. Ir. J. Med. Sci..

[13] Kameda-Smith M.M., Iorio-Morin C., Winkler-Schwartz A., Ahmed U.S., Bergeron D., Bigder M., Dakson A., Elliott C.A., Guha D., Lavergne P. (2018). Smartphone Usage Patterns by Canadian Neurosurgery Residents: A National Cross-Sectional Survey. World Neurosurg..

[14] Terry D.L., Terry C.P. (2018). Smartphone Use and Professional Communication among Medical Residents in Primary Care. PRiMER.

[15] Gipson S., Torous J., Boland R., Conrad E. (2017). Mobile phone use in psychiatry residents in the United States: Multisite cross-sectional survey study. JMIR mHealth uHealth.

[16] Nerminathan A., Harrison A., Phelps M., Scott K.M., Alexander S. (2017). Doctors’ use of mobile devices in the clinical setting: A mixed methods study. Intern. Med. J..

[17] Stergiannis P., Intas G., Toulia G., Tsolakoglou I., Kostagiolas P., Christodoulou E., Chalari E., Kiriakopoulos V., Filntisis G. (2017). Clinical use of smartphones among medical and nursing staff in Greece: A survey. Comput. Inform. Nurs..

[18] Vallangeon B.D., Hawley J.S., Sloane R., Bean S.M. (2017). An assessment of pathology resident access to and use of technology: A nationwide survey. Arch. Pathol. Lab. Med..

[19] Jahanshir A., Karimialavijeh E., Vahedi H., Momeni M. (2017). Smartphones and medical applications in the emergency department daily practice. Emergency.

[20] Kilber J., Barclay A.C., Ohmer D.G. (2014). Seven Tips for Managing Generation Y. J. Manag. Policy Pract..

[21] Nakagawa K., Yellowlees P. (2020). Inter-generational Effects of Technology: Why Millennial Physicians May Be Less at Risk for Burnout Than Baby Boomers. Curr. Psychiatry Rep..

[22] Moreira A., Portela F., Santos M. (2019). Bring Your Own Device in Healthcare. Int. J. Priv. Health Inf. Manag..

[23] Bromwich M., Bromwich R. (2016). Privacy risks when using mobile devices in health care. CMAJ.

[24] Thomairy N.A., Mummaneni M., Alsalamah S., Moussa N., Coustasse A. (2015). Use of Smartphones in Hospitals. Health Care Manag..

[25] Hedström K., Kolkowska E., Karlsson F., Allen J.P. (2011). Value conflicts for information security management. J. Strateg. Inf. Syst..

[26] Klick J., Koch R., Brandstetter T. Epidemic? The Attack Surface of German Hospitals during the COVID-19 Pandemic. Proceedings of the 2021 13th International Conference on Cyber Conflict (CyCon).

[27] Hu Q., Dinev T., Hart P., Cooke D. (2012). Managing Employee Compliance with Information Security Policies: The Critical Role of Top Management and Organizational Culture. Decis. Sci..

[28] D’Arcy J., Greene G. (2014). Security culture and the employment relationship as drivers of employees’ security compliance. Inf. Manag. Comput. Secur..

[29] Solomon G., Brown I. (2020). The influence of organisational culture and information security culture on employee compliance behaviour. J. Enterp. Inf. Manag..

[30] Humpert-Vrielink F., Graf D., Henke V., Hülsken G., Meier P.-M., Beß A. (2022). Daten, Informationen und Informationssicherheit: Theorie und Praxis. Digitalstrategie im Krankenhaus.

[31] Semmelmayer H. (2022). IT-Sicherheit 2022: Was PDSG und KRITIS 2.0 für Kliniken bedeuten. Klin. Manag. Aktuell.

[32] Burr H., Berthelsen H., Moncada S., Nübling M., Dupret E., Demiral Y., Oudyk J., Kristensen T.S., Llorens C., Navarro A. (2019). The Third Version of the Copenhagen Psychosocial Questionnaire. Saf. Health Work.

[33] Lincke H.-J., Vomstein M., Lindner A., Nolle I., Häberle N., Haug A., Nübling M. (2021). COPSOQ III in Germany: Validation of a standard instrument to measure psychosocial factors at work. J. Occup. Med. Toxicol..

[34] Keller M., Bamberg E., Kersten M., Nienhaus A. (2010). Entwicklung eines Instruments zur stressbezogenen Arbeitsanalyse für Klinikärztinnen und -ärzte (ISAK). Z. Arb. Wiss..

[35] Keller M., Bamberg E., Kersten M., Nienhaus A. (2013). Validierung des Instruments zur stressbezogenen Arbeitsanalyse für Klinikärztinnen und -ärzte (ISAK). Z. Arb. Organ..

[36] Karlsson F., Karlsson M., Åström J. (2017). Measuring employees’ compliance—The importance of value pluralism. ICS.

[37] Cohen J. (1988). Statistical Power Analysis for the Behavioral Sciences.

[38] Wessel D., Attig C., Franke T., Alt F., Bulling A., Döring T. (2019). ATI-S—An Ultra-Short Scale for Assessing Affinity for Technology Interaction in User Studies. Proceedings of the Mensch und Computer 2019.

[39] Wani T.A., Mendoza A., Gray K. (2020). Hospital Bring-Your-Own-Device Security Challenges and Solutions: Systematic Review of Gray Literature. JMIR Mhealth Uhealth.

[40] Krey M. (2018). A framework for the adoption of bring your own device (BYOD) in the hospital environment. GSTF J. Comput..

[41] Kellerman S. (2001). Physician response to surveys A review of the literature. Am. J. Prev. Med..

[42] Schouten B., Cobben F., Bethlehem J. (2008). Indicators for the Representativeness of Survey Response. Surv. Methodol..

[43] Ortmanns V., Schneider S.L. (2016). Can we assess representativeness of cross-national surveys using the education variable?. Surv. Res. Methods.

